# Effect of Aso limonite on anaerobic digestion of waste sewage sludge

**DOI:** 10.1186/s13568-020-01010-w

**Published:** 2020-04-16

**Authors:** Nurul Asyifah Mustapha, Shotaro Toya, Toshinari Maeda

**Affiliations:** Department of Biological Functions Engineering, Graduate School of Life Science and Systems Engineering, Kyushu Institute of Technology, 2-4 Hibikino, Wakamatsu-ku, Kitakyushu, 808-0196 Japan

**Keywords:** Limonite, Volcanic residue, Methane inhibition, Hydrogen sulfide inhibition, Ammonia inhibition

## Abstract

The effect of Aso volcanic limonite was explored in anaerobic digestion using waste sewage sludge (WSS). In this study, methane and hydrogen sulfide were remarkably inhibited when Aso limonite was mixed with WSS as well as a significant reduction of ammonia. Although pH was lowered after adding Aso limonite, methane was still inhibited in neutralized pH condition at 7.0. Hydrolysis stage was not influenced by Aso limonite as supported by the result that a high protease activity was still detected in the presence of the material. However, acidogenesis stage was affected by Aso limonite as indicated by the different productions of organic acids. Acetic acid, was accumulated in the presence of Aso limonite due to the inhibition of methane production, except in the highest concentration of Aso limonite which the production of acetate may be inhibited. Besides, the production of propionate and butyrate reduced in accordance to the increased concentration of Aso limonite. In addition, Archaeal activity (methanogens) in WSS with Aso limonite was low in agreement with the low methane production. Thus, these results indicate that Aso limonite influences the acidogenesis and methanogenesis processes, by which the productions of methane and ammonia were inhibited. On the other hand, in the contactless of Aso limonite during the anaerobic digestion of WSS (Aso limonite was placed in the area of headspace in the vial), Aso limonite had the adsorptive ability for hydrogen sulfide from WSS, but not for methane. This contactless system of Aso limonite may be a practical means to remove hydrogen sulfide without inhibiting methane production as an important bioenergy source.

## Introduction

The activated sewage process is one of the most important wastewater treatment systems capable of removing organic compounds from any wastewater (Modin et al. 2015). In general, the process is handled aerobically to promote the degradation or the uptake of organic matter by enhancing the functions of aerobic bacteria in sewage sludge (Shchegolkova et al. 2016). On the other hand, a large amount of bacterial aggregates called excess sewage sludge are produced daily by the process and the extra sewage sludge is treated as an industrial waste (Maeda et al. 2009). To date, more than 70% of the waste sewage sludge (WSS) has been reported to be recycled (Kacprzak et al. 2017); for example, anaerobic digestion of WSS is one of the major approaches to recycle WSS because methane gas should be produced as bioenergy from this process (Weiland 2010). However, hydrogen sulfide and ammonia which are basically produced as by-products during the anaerobic digestion of WSS has a corrosive effect on the fermentation tank (Li et al. 2019). In addition, these odorous gases are harmful to humans through direct irritations or psychopathologic mechanisms (Schiffman and Williams 2005) and have a negative impact on methane fermentation as a high concentration of hydrogen sulfide lowers the quality of biogas production (Zhou et al. 2016) as well as the ammonia triggers to accumulate the organic acids (Wu et al. 2016). Therefore, the removal of hydrogen sulfide and ammonia during the anaerobic digestion of WSS is essential to the long-term operation of anaerobic digestion (Dai et al. 2017).

To date, various strategies in mitigating the production of malodorous substances such as hydrogen sulfide and ammonia have been carried out. For example, precipitation methods using metal salts (He et al. 2009), oxidation method using some oxidizing reagents such as potassium permanganate and hydrogen peroxide (McCrory and Hobbs 2001), and adsorptive treatments using activated carbon and zeolite (Wheeler et al. 2011; Zulkefli et al. 2019) are known as a means to remove hydrogen sulfide. In addition, a stripping process (Yuan et al. 2016) and a biological process using ammonia oxidizing bacteria (Ding et al. 2019) have been studied to remove ammonia. Most of the studies focus on enhanced methane production using WSS. In particular, the effect of some additives such as chemical reagents is well studied; for example, azithromycin and the analogs improve methane fermentation (Mustapha et al. 2018) and nano zero-valent iron material increases the yield of methane by reducing the toxicity of phenolic compounds (Dong et al. 2019). Besides, some pretreatments of WSS have been studied for enhancement of methane production. For instance, the thermal-alkaline (Liu et al. 2019) and nitrite-based (Liu et al. 2020) pretreatments have assisted the disruption of sludge flocs thus improved the release of biodegradable organic matter as substrates to be further utilized in anaerobic digestion process, in which methane production can be improved. In addition, some studies report that some of the Gram-positive bacteria may have a negative impact on methane fermentation using WSS. This has been shown by the addition of AiiM lactonase, a quorum quenching enzyme that has inhibited methane production, whereas the percentage of Gram-positive bacteria was increased in WSS (Nguyen et al. 2019). Besides, a pretreatment of WSS using sulfite has led to the destruction of Gram-positive bacteria, thereby at the same time methane production was improved (Zan et al. 2019). Therefore, these studies may provide a new hint to improve methane fermentation using WSS. On the other hand, there are a very limited number of studies describing an efficient removal of hydrogen sulfide. A recent study indicates that ferric oxide and ferric citrate are effective to reduce the production of hydrogen sulfide without inhibiting methane production (Jiang et al. 2017); therefore, these iron materials may be useful for constructing an eco-friendly technology capable of enhancing methane production and removing malodorous substances. However, for the practical usage of these iron materials, a cheap material as much as possible must be selected from the aspect of material cost (Tsubouchi et al. 2008).

Limonite, a natural resource obtained from Aso Mountain in Kumamoto Prefecture, Japan is one of the cheapest iron materials, which is worthy to test methane fermentation using WSS. This limonite is originally formed from the magmas that were erupted from the volcanos. The groundwater that is rich in ferrous iron moves to the ground surface and deposits limonite. The detail compositions of limonite obtained from Aso are shown in Table 1. In Indonesia, limonite, previously called as iron oxyhydroxides has a prolonged contact with seawater. The high pH of seawater cause a progressive hydrolysis and dissolution of SiO_2_, which consequently leads to the enrichment of Fe_2_O_3_ in limonite accounted for 68.8% (Sturesson et al. 2000). As mentioned in their paper, iron oxyhydroxides have a large surface area, which favours the adsorption of phosphate, aluminium, and other cations. Besides, a study by Li et al. (2007) used an Indonesian natural limonite with 41.4% iron content as an iron-based catalyst for light fuel gas production from coal volatile decomposition. On the other hand, one research from China that obtained a natural iron ore also called as limonite from a geological specimen factory. Five iron ores including limonite have a certain effect to reduce the amount of hydrogen sulfide (Zhou et al. 2016); however, the comprehensive evaluation to use limonite for the production of not only hydrogen sulfide but also methane and ammonia is not done anything yet.

**Table 1:** Inorganic and organic compositions of limonite from Aso Mountain. Source: Japan Limonite Co., Ltd

Compositions	Percentage % (g/kg)	Compositions	Percentage % (g/kg)
Iron—Fe_2_O_3_	6.908	Ash	7.480
Silica—SiO_2_	1.370		
Aluminium—Al_2_O_3_	0.276	Water	1.380
Calcium—CaO	0.149		
Sulphur—S	0.058	Carbohydrate	1.060
Magnesium—MgO	0.051		
Potassium—K	0.020	Protein	0.060
Phosphorus—P	0.009		
Manganese—Mn	0.003	Fat	0.010
Sodium—Na	0.002		

Hence, in this study, the effect of Aso limonite on the anaerobic digestion was investigated by using WSS. In detail, methane and malodorous gases (hydrogen sulfide and ammonia) as well as microbial community composition were evaluated in the presence of limonite.

## Materials and methods

### Limonite

Limonite used in this study was a natural material produced from the volcanic eruption of Aso Mountain in Kumamoto Prefecture, Japan. It was kindly provided by Japan Limonite Co. Ltd. The limonite was directly used for methane fermentation and other analyses.

### Preparation of waste sewage sludge

WSS was acquired from the secondary treatment stage of Hiagari Wastewater Treatment Plant in Kitakyushu City, Japan. Prior to the experiments, the WSS was washed three times using distilled water by the centrifugation at 8000×*g* for 10 min at 4 °C to remove the initial supernatant containing endogenous compounds. The remaining pellet was resuspended in distilled water by vigorous shaking to be the final concentration of 10% (wet sludge [w/w]). The total solids (TS), volatile solids (VS), and pH of WSS were 6.91 ± 0.02 mg/L, 6.01 ± 0.01 mg/L, and 7.2 ± 0.1, respectively.

### Methane and hydrogen sulfide assays using limonite

Methane assay was performed in three ways to evaluate methane production and microbial activity in WSS with or without limonite or another iron material as follows. (1) Limonite was mixed with WSS inside a vial. WSS and different concentrations of limonite (0.5–10% w/v) were placed into 66-mL vials to make 30 mL of the total volume. Because the pH of WSS samples was lowered to be around pH 4 by adding limonite, the initial pH was adjusted at pH 7 for all the samples with limonite. Then, two control WSS samples were prepared to compare the effect of the initial pH decrease by limonite: one control WSS was prepared to be pH 7 using 1 M NaOH and the other control WSS was initially treated to be pH 4 using 1 M HCl and then adjusted at pH 7 using 1 M NaOH. (2) To investigate the effect of a limonite composition; 5% (w/v) of iron (III) oxide (Fe_2_O_3_) which is rich in limonite was used in place of limonite and added into 30 mL of WSS. (3) Limonite was place at the top side of a 66-mL vial hold with a sponge so as not to be mixed with WSS (30 mL). All the vials prepared were tightly sealed using butyl rubber stoppers, crimped, and sparged with nitrogen gas for 2 min to create anaerobic conditions. The vials were then incubated at 37 °C and shaken at 120 revolutions per minute (rpm) for about 2 weeks. Each experiment was conducted at least in triplicate.

Methane was measured by injecting 50 µL of headspace gas from the vials into a GC-3200 gas chromatograph (GL Science, Japan) equipped with a thermal conductivity detector and a column of Molecular Sieve 13 × 60/80 mesh column, SUS 2 × 3 mm I.D (GL Science, Japan). Helium gas was used as a carrier gas (40 mL/min). The gas chromatography conditions were as follows: current, 100 mA; oven, injector, and detector temperatures, 40 °C, 50 °C, and 65 °C, respectively.

In addition, hydrogen sulfide was measured using the GASTEC system which is a gas sampling pump set with pump stroke counter. The hydrogen sulfide detector tube of different ranges ppm was attached to this system and to the vial using syringe to detect the hydrogen sulfide produced in the headspace of the vials. Both gases were calculated based on volatile solid (VS) of WSS (with or without limonite).

### Analytical methods

WSS samples during the fermentation were used for the following analyses: pH, protein concentration, protease activity, organic acids, and ammonia. Initially, WSS samples were centrifuged at 13,000 rpm for 7 min to collect the supernatants, which were then filtered through a 0.2 µm membrane syringe filter. Then, pH was measured using a compact pH meter (AS ONE, AS-211, Japan). The soluble protein concentration was analysed using the Lowry method with bovine serum albumin as a standard (Lowry et al. 1951). Protease activity was measured as described previously (Maeda et al. 2011). One unit of protease activity was defined as the quantity of tyrosine (mmol) produced from casein per minute by 1 mg of enzyme. Organic acids were analysed using high-performance liquid chromatography (Shimadzu LC-10AD) as described previously (Mohd Yusoff et al. 2012). Ammonia concentration in WSS supernatant was measured using ammonia assay kit (Wako, Japan) (Inokuma et al. 2018). Each assay was conducted at least in triplicate.

### RNA extraction and cDNA synthesis

Total ribonucleic acid (RNA) was extracted using the RNeasy kit (Qiagen, Valencia, CA) as described previously (Mohd Yusoff et al. 2012). The complementary deoxyribonucleic acid (cDNA) was synthesized using the PrimeScript RT Reagent Kits (TAKARA Bio Inc., Shiga, Japan) as described previously (Mustapha et al. 2016). The cDNA was used as a template to identify bacterial and archaeal population using quantitative real-time polymerase chain reaction (qRT-PCR) and bacterial communities using MiSeq.

### qRT-PCR and high-throughput of 16S rRNA gene sequencing

The qRT-PCR quantification for bacteria and archaea was performed by using StepOne Real Time PCR System (Applied Biosystem) for amplification and detection of fluorescence by specific primers and probes of TaqMan system. The real-time PCR mixture and cycling conditions were explained in details previously (Mustapha et al. 2017). Besides, high-throughput sequencing target 16S rRNA gene sequence selected from V3–V4 region using forward primer, 341F (5′-CCTACGGGNGGCWGCAG-3′) and reverse primer, 785R (5′-GACTACHVGGGTATCTAATCC-3′) (Klindworth et al. 2013). The sequencing was based on the protocol for 16S metagenomics sequencing library preparation provided by Illumina MiSeq system and was described previously (Mustapha et al. 2018). The high-throughput data analysis was done using LotuS pipeline (Hildebrand et al. 2014) to process the demultiplexed raw paired-end reads sequences and then classified into different taxonomic levels as previously described in details (Mustapha et al. 2018). All raw sequence data were deposited into NCBI Sequence Reads Archive (SRA) database under the Accession number of SRP072534.

### Statistical analysis

Different samples were compared with control WSS using means from at least triplicate data (n = 3). Comparison was performed using means and standard deviations by the Student’s t test (GraphPad software) at a significance level of p < 0.05.

## Results

### Effect of limonite on the productions of methane and malodorous substances

**Fig. 1:**
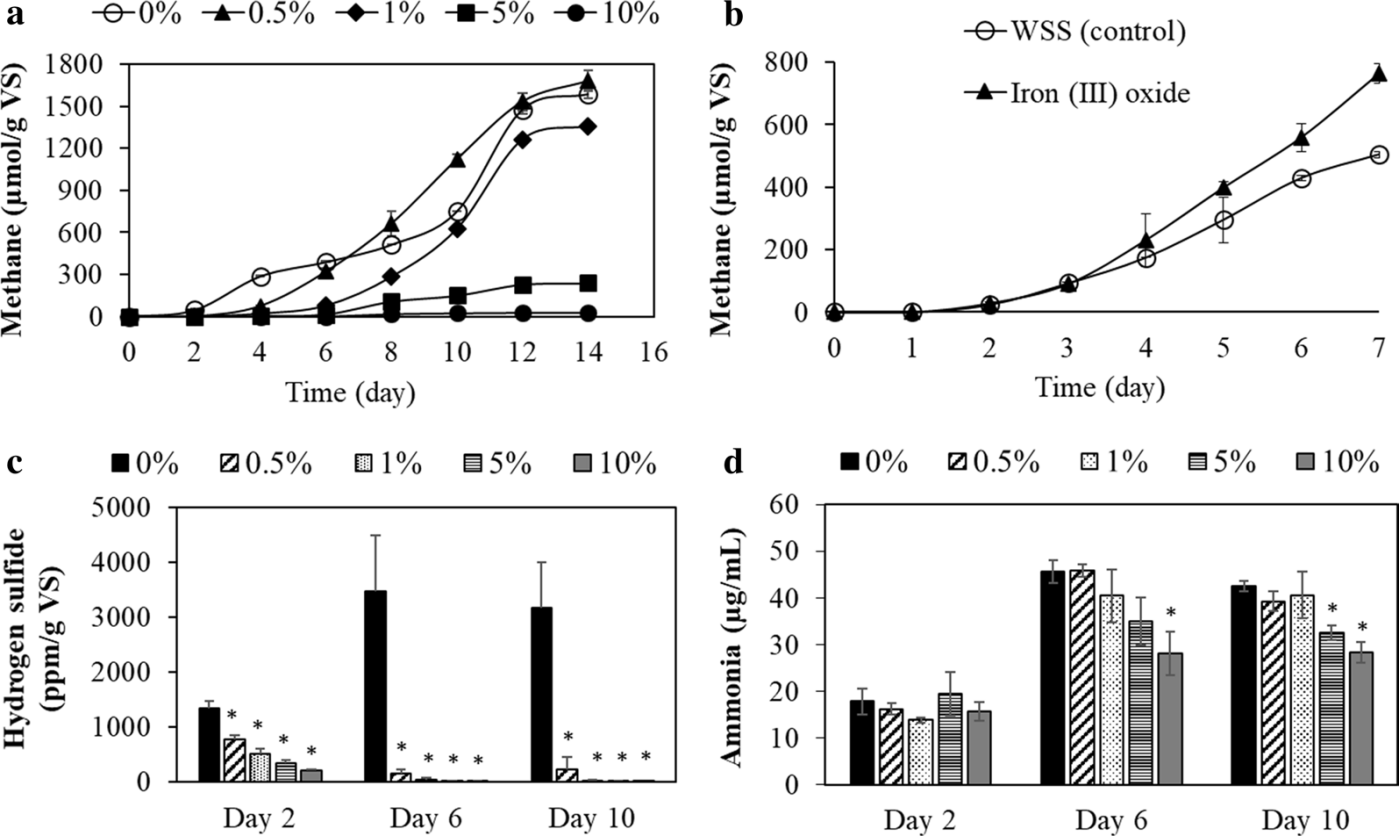
Impact of Aso limonite on the production of gases (methane and hydrogen sulfide) and ammonia in anaerobic digestion processes using waste sewage sludge (WSS); **a** methane production in different percentage of Aso limonite, **b** methane production in the WSS with iron (III) oxide at 5% (w/v), **c** hydrogen sulfide and **d** ammonia were measured at day 2, 6, and 10 of incubation in different percentage of Aso limonite. The legend denotes the concentration of Aso limonite as % (w/v). Error bars represent the standard errors (n = 3). Asterisk (*) indicate the significant difference by the addition of Aso limonite

First, the effect of limonite during the anaerobic digestion was investigated by evaluating the productions of methane, hydrogen sulfide, and ammonia. Prior to the anaerobic digestion process, the addition of 0.5%, 1%, 5%, and 10% (w/v) limonite lowered the pH of WSS samples to pH 6.0, pH 5.4, pH 4.1, and pH 4.0, respectively. Therefore, the pH of all the samples was initially adjusted at around pH 7 before the incubation. Methane production was recorded during 2 weeks of incubation as shown in Fig. 1a. Lower concentrations of limonite (0.5% w/v and 1% w/v) had a slight inhibitory effect at an early stage on the methane production, as the amounts of methane were almost comparable with the control WSS and 0.5% (w/v) of limonite showed slightly higher methane production than the control after 8 days. On the other hand, at higher concentrations of limonite (5% and 10% w/v), methane production was remarkably inhibited. WSS with 5% (w/v) of limonite reduced the methane production by sixfold as compared to the control WSS at the end of fermentation, recorded as 237 ± 46 vs 1587 ± 31 µmol/g VS. The highest concentration of limonite used in this study was 10% (w/v) which totally inhibited the methane production from WSS. Next, since iron (III) oxide (Fe_2_O_3_) is a main component of limonite, the effect of Fe_2_O_3_ on anaerobic digestion was tested. As a result, the addition of 5% (w/v) of Fe_2_O_3_ enhanced the production of methane (Fig. 1b) unlike Aso limonite which inhibited methane production. Interestingly, the initial pH of WSS was not changed by the addition of iron (III) oxide whereas the addition of Aso limonite triggered acidic conditions to be around pH 4.

On the other hand, hydrogen sulfide was also produced from the anaerobic digestion of WSS. As shown in Fig. 1c, hydrogen sulfide was detected at 2 days in all the WSS samples with or without limonite. However, the amount of hydrogen sulfide reduced in the presence of limonite. Interestingly, at 6 and 10 days, hydrogen sulfide was almost completely removed in all the WSS samples with limonite whereas a high amount of hydrogen sulfide was detected at the control sample in the absence of limonite.

Furthermore, ammonia was measured during the anaerobic digestion as shown in Fig. 1d. Because gaseous ammonia was not detected during the fermentation, dissolved ammonia was monitored with time. The total ammonia started to accumulate after 6 days but showed reduction with the addition of high Aso limonite.

### Characteristics of Aso limonite and its effect on anaerobic digestion

**Fig. 2:**
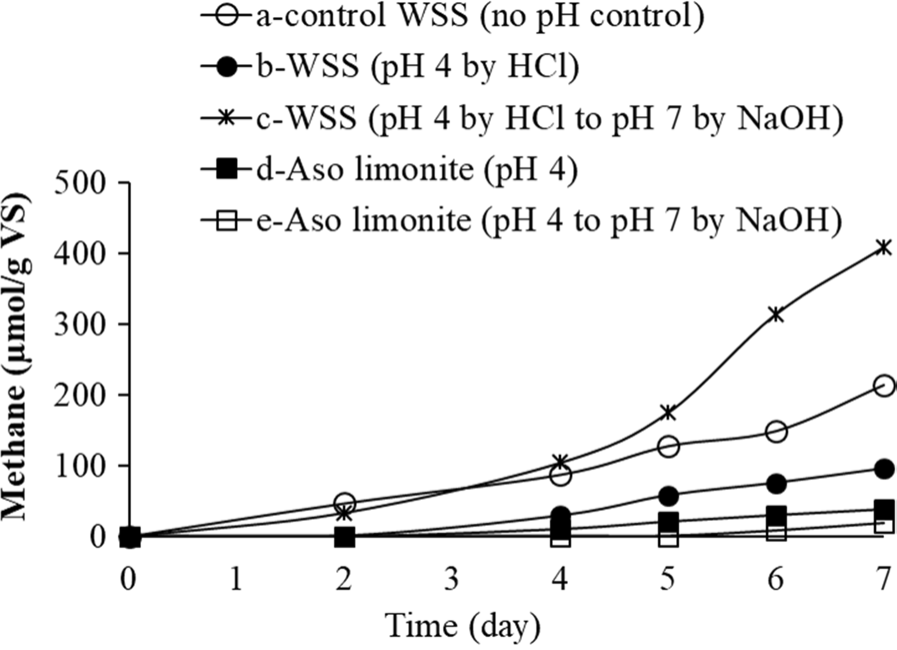
pH effect in the inhibition of methane fermentation by Aso limonite. Since the addition of Aso limonite triggered to be at around pH 4, the following five WSS samples were prepared for the anaerobic digestion for 7 days: a—control WSS (no pH control), b—the pH of WSS was initially adjusted at pH 4 by adding HCl, c—the WSS at pH 4 was further readjusted at pH 7 by adding NaOH, d—Aso limonite was added to WSS, and e—the WSS with Aso limonite was readjusted at pH 7 by adding NaOH. The concentration of Aso limonite used in this experiment was 5% (w/v). Error bars represent the standard errors (n = 3)

It was mentioned earlier that the addition of Aso limonite has decreased the pH condition in WSS. Since pH is one of the reasons to change microbial activity in WSS, the effect of initial pH decrease by limonite on methane fermentation was investigated. Therefore, WSS samples were prepared and examined to see the effect of pH change as follows: (a) control WSS was prepared at pH 7, (b) pH of WSS was adjusted at pH 4, (c) pH of WSS was initially adjusted at pH 4 then adjusted at pH 7, (d) pH of WSS was changed at pH 4.3 by adding 5% (w/v) Aso limonite, (e) WSS mixed with 5% (w/v) Aso limonite was adjusted from pH 4.3 to pH 7. As shown in Fig. 2, contrast to the control WSS (sample a), almost no methane was detected in WSS with limonite adjusted at pH 7 (sample e). In the sample c, methane production increased after 5 days without inhibiting it, indicating that the initial pH decrease may facilitate the hydrolytic reactions. Regardless of pH adjustment, methane production was much lower and almost completely inhibited in the presence of limonite during the anaerobic digestion for 7 days.

**Fig. 3:**
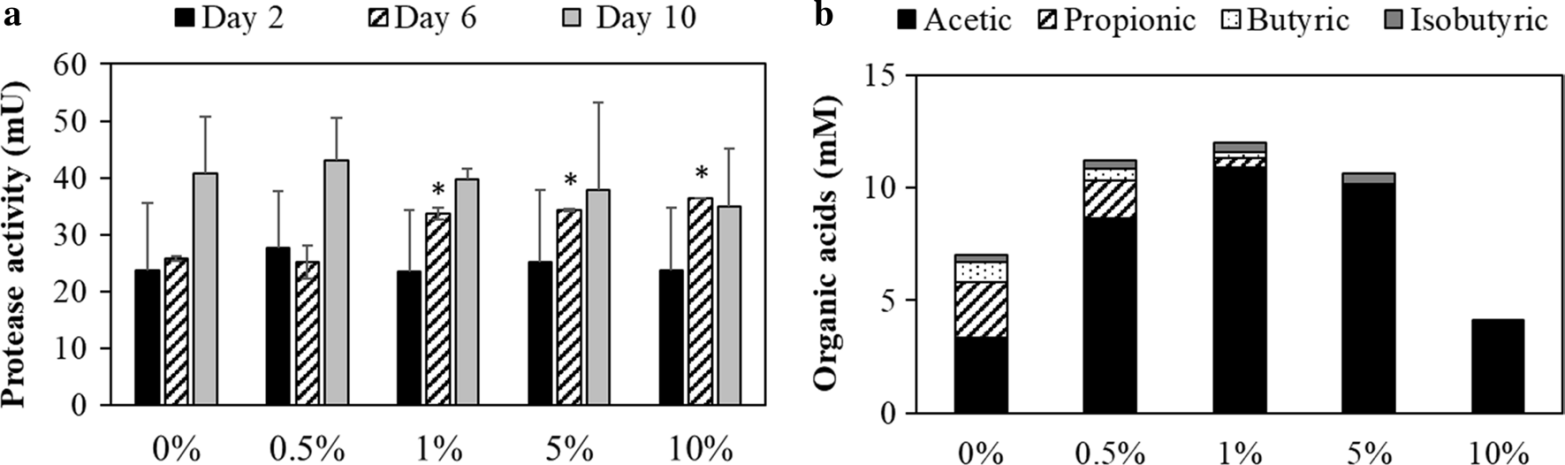
Impact of Aso limonite to the hydrolysis and the acidogenesis stages of anaerobic digestion; **a** protease activity at day 2, 6, and 10 to evaluate the hydrolytic activity of WSS and **b** organic acids at day 6 to evaluate the acidogenesis process. Aso limonite was mixed with WSS to be the following concentrations (0%, 0.5%, 1%, 5%, and 10% w/v). Error bars indicate standard errors (n = 3). Asterisk (*) indicate the significant difference by the addition of Aso limonite

Next, to clarify the inhibitory mechanism for methane production by Aso limonite, hydrolysis and acidogenesis stages during the methane fermentation using WSS were evaluated. At the hydrolysis stage, large molecules such as proteins, carbohydrates and lipids are converted into small molecules by hydrolytic enzymes such as proteases, amylases, cellulases, and lipases. Since protein is the main component of WSS (Maeda et al. 2009), dynamics of protein concentration and protease activity were monitored to evaluate this hydrolysis stage. The protein concentration was slightly high at 5% and 10% of limonite at day 2. At day 6 and 10, no significant difference of protein concentration was observed between control WSS and WSS with limonite (data not shown). On the other hand, as shown in Fig. 3a, a slightly higher protease activity was detected at 1%, 5% and 10% (w/v) of limonite at day 6. In addition, Fig. 3b shows the profile of organic acids produced from WSS samples with or without Aso limonite after 6 days to evaluate the stage of acidogenesis. Acetic, propionic, butyric, and isobutyric acids were the main organic acids detected in these samples. A high concentration of acetic acid was detected in all samples as compared to other organic acids. However, the concentration of acetic acid was lowest in the addition of 10% (w/v) of Aso limonite as compared to other concentration of Aso limonite in WSS. Although acetic acid production was low in control WSS, other organic acids were present at higher concentration. Particularly, propionic acid was only produced in control WSS and in the WSS samples at 0.5% and 1% (w/v) of limonite. Similarly, the production of butyric acid also was recorded in control WSS samples and in the WSS samples with low percentage of Aso limonite whereas isobutyric acid was slightly produced in all the samples at day 6 of anaerobic digestion.

### Influence of Aso limonite on microbial activity and community

**Fig. 4:**
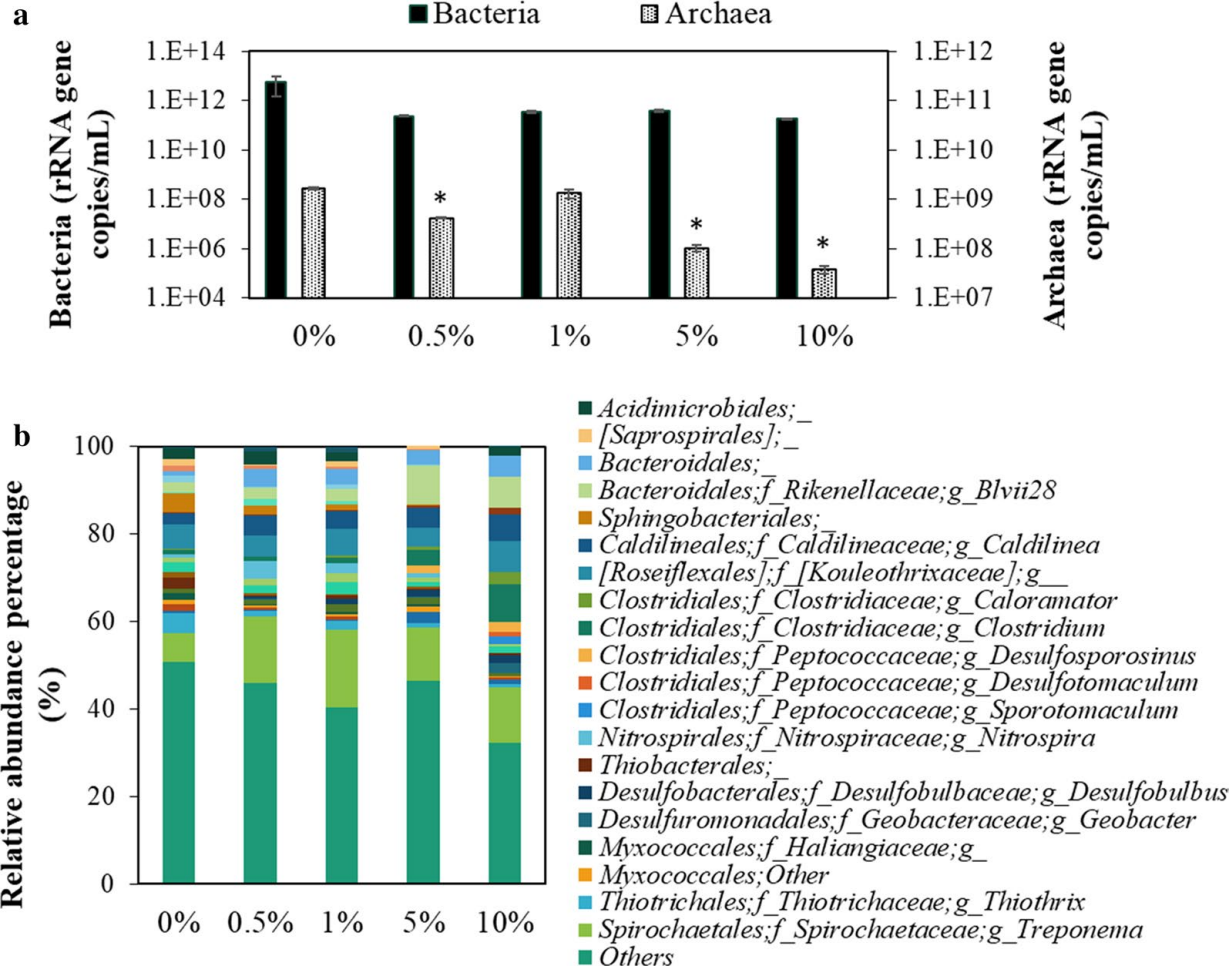
Impact of Aso limonite to bacterial/archaeal activities and bacterial community in WSS at the end of anaerobic digestion process; **a** proportion of active bacteria and archaea determined by the quantitative real-time PCR and **b** relative abundant percentages of dominant bacterial community indicated by Order, Family, and Genus level (if available). Error bars indicate standard errors (n = 3). Asterisk (*) indicate the significant difference by the addition of Aso limonite

Furthermore, the changes of microbial activity and community were evaluated to see the impact of Aso limonite to the microbes in WSS. For this study, RNA was used as a template for the analyses of microbial activity and bacterial community so that really-active microorganisms can be evaluated in the WSS samples with or without Aso limonite. First, a qRT-PCR reaction targeting bacteria or archaea was performed to evaluate the active bacterial or archaeal population. As shown in Fig. 4a, the highest population of archaea was detected in the control WSS recorded as 1.65 ± 0.08 × 10^9^ rRNA gene copies/mL and almost the same numbers of archaea were present in the WSS containing 0.5% and 1% (w/v) of Aso limonite. In contrast, the lowest number of archaeal population (3.7 ± 0.6 × 10^7^ rRNA gene copies/mL) was recorded at 10% (w/v) of Aso limonite. Besides, the number of bacterial population was reduced in all WSS samples with the addition of Aso limonite.

Next, 16S metagenomics analysis was carried out using MiSeq sequencer to investigate the details of bacterial community. Figure 4b shows the bacterial community of WSS with or without Aso limonite. In control WSS without Aso limonite, the major bacterial community present in the order level of taxonomy are *Thiotrichales*, *Thiobacterales*, and *Sphingobacteriales*. On the other hand, by the addition of limonite, *Clostridiales*, *Desulfuromonadales*, *Desulfobacterales*, *Bacteroidales*, and *Spirochaetales* are detected at relatively-high abundant ratios as the bacterial community.

### Adsorption of hydrogen sulfide by Aso limonite

**Fig. 5:**
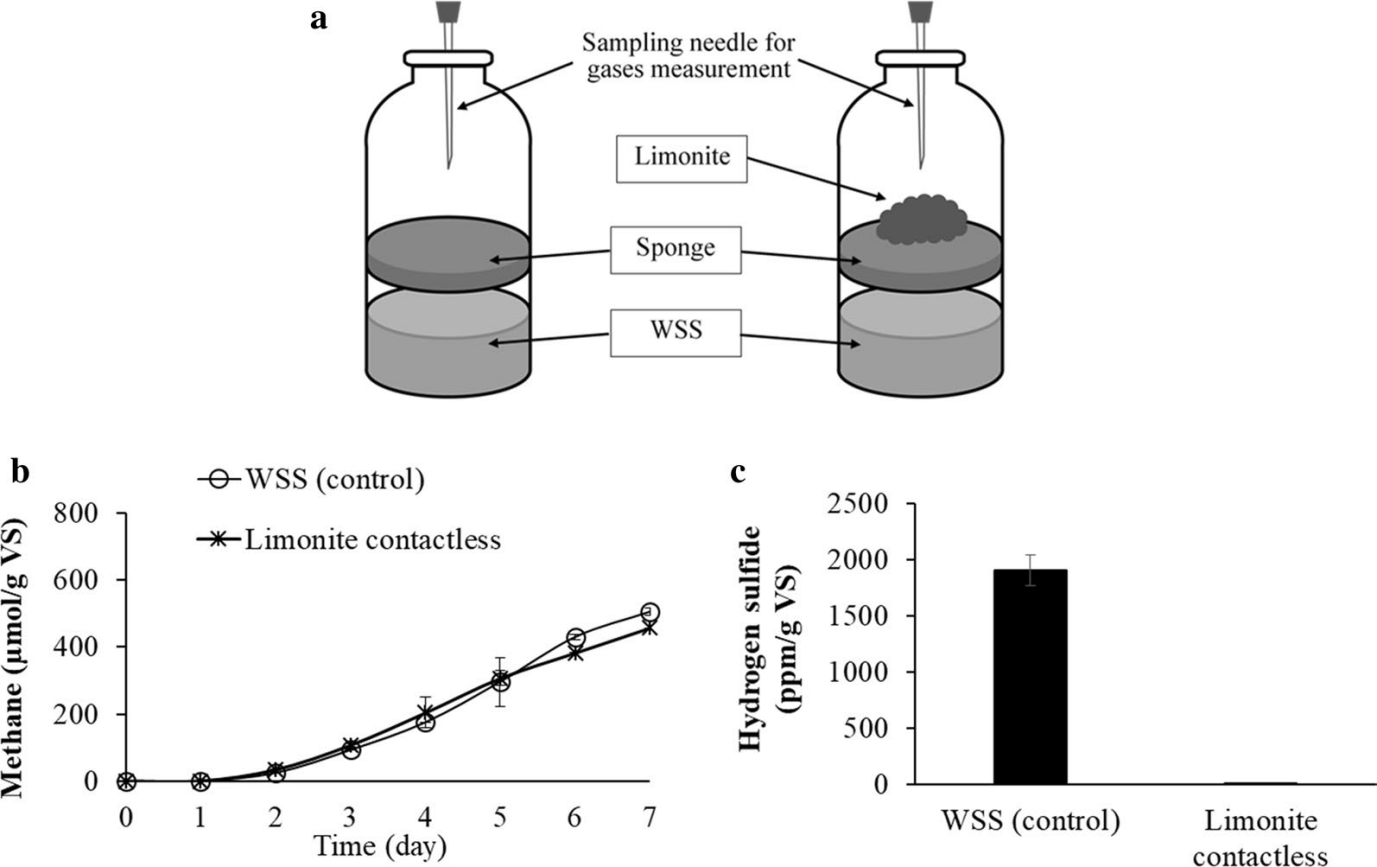
Anaerobic digestion by a contactless system of WSS with or without Aso limonite. **a** A contactless system was constructed as illustrated. Two vials were prepared: (left) Aso limonite cannot be used for the anaerobic digestion and (right) Aso limonite cannot be mixed with WSS by separately placing it on a sponge. **b** Methane production and **c** hydrogen sulfide production from control WSS and with contactless limonite. The concentration of Aso limonite used in this experiment was 5% (w/v). Error bars represent the standard errors (n = 3)

A reliable capability of Aso limonite to inhibit the production of hydrogen sulfide is desirable; however, the effect to methane production is not suitable because methane is one of the bioenergy resources (Weiland 2010). Therefore, a practical usage of Aso limonite for the anaerobic digestion using WSS was examined by using the system shown in Fig. 5a. In this system, Aso limonite and WSS were separately set inside a vial by using a sponge material. In the control vial without the limonite, methane and hydrogen sulfide were produced with time. Aso limonite did not trap methane produced from WSS as shown in the result that the amounts of methane detected were the same with or without Aso limonite (Fig. 5b). On the other hand, a very low amount of hydrogen sulfide was detected in the presence of limonite set separately inside the vial. The exact amount of hydrogen sulfide was 1.6 ± 0.4 ppm/g VS in the presence of Aso limonite whereas the control WSS without limonite recorded 1904 ± 136 ppm/g VS of hydrogen sulfide after 7 days as shown in Fig. 5c. In addition, other measurements including protease activity, ammonia, and organic acids production showed no significant difference (data not shown) between WSS in contactless system with or without Aso limonite.

## Discussion

### Inhibition of methane and malodorous substances by Aso limonite

The production of methane from WSS with the addition of Aso limonite was inhibited in a dose-dependent manner. However, the evaluation of methane production using Fe_2_O_3_ as the main composition of Aso limonite showed oppositely. Our result using Fe_2_O_3_ was similar to a study that utilized FeCl_3_ in anaerobic bioconversion of dairy manure for the enhancement of biogas production (Lar and Xiujin 2009). On the other hand, there are some inconsistent reports with us: a study by Jäckel and Schnell (2000) using ferric iron suppressed the methane production from paddy rice field and a study by Van Bodegom et al. (2004) reported the effect of amorphous Fe(OH)_3_ which directly inhibited methane production by influencing a few species of methanogens. Thus, a reason to contradict the effect of iron compounds on anaerobic digestion may be due to the difference of microbial consortia in each microbial source, which can be affected by iron compounds or due to the difference of iron compounds.

In addition, an iron-rich compound has been speculated to inhibit the production of hydrogen sulfide to improve anaerobic digestion process. In the bioenergy production, it has been reported that hydrogen sulfide (0.1–3%) triggers to diminish biogas production or produce a low quality of biogas (Lar and Xiujin 2009). Another literature indicates that limonite reduces the amount of hydrogen sulfide; however, the production of biogases including methane is not influenced by the limonite material (Zhou et al. 2016). Furthermore, a study by Jiang et al. (2017) showed that various iron compounds such as FeCl_2_, FeCl_3_, Fe(OH)_3_, and Fe_2_O_3_ have a certain ability to reduce the emission of hydrogen sulfide from chicken manure without affecting methane production. Conversely, in this study, the gases, hydrogen sulfide and methane reduced in the presence of limonite. The difference of results between Aso limonite and other limonite is still unknown; however, a site-dependent composition of limonite in Aso may be a causal factor for the inconsistent data. Importantly, the ammonia concentration reduced in a dose-dependent manner of limonite. The low pH triggered by the addition of Aso limonite may be led to the reduction of ammonia as mentioned in a previous report that the acidification process can mitigate the emission of ammonia from animal waste (Dai and Blanes-Vidal 2013). Thus, Aso limonite may be a potent material capable of reducing the productions of not only methane and hydrogen sulfide but also dissolved ammonia although the removal efficiency of ammonia was quite low compared to that of methane or hydrogen sulfide.

### Inhibitory mechanisms of Aso limonite in anaerobic digestion

It is important to understand the inhibitory mechanism of limonite for the productions of methane and hydrogen sulfide; therefore, we hypothesized the following two possibilities: the inhibitory effect may be due to (1) the change of microbial activity through the addition of Aso limonite or (2) the adsorptive activity of Aso limonite. The changes of pH is one of the reasons for microbial activity changes. Based on our findings, the initial pH decrease by Aso limonite may be not a main reason on low methane production in the presence of Aso limonite because a very low methane production was observed in the sample e in which the pH was neutralized to pH 7 (Fig. 2). In the presence of limonite, methane production was much lower and almost completely inhibited during the anaerobic digestion. This indicates that besides low pH, limonite itself may have the ability to directly inhibit methane production. An optimal pH for methane production through anaerobic digestion has been reported to be in the range of pH 6.8–7.2 and the growth of methanogens responsible for methane production will reduced at less than pH 6.6 (Ward et al. 2008).

The inhibitory mechanisms for methane production by Aso limonite was clarified on hydrolysis and acidogenesis stages during the methane fermentation using WSS. At the hydrolysis stage, large molecules of proteins are converted into smaller molecules by hydrolytic proteases. It suggests that during the anaerobic digestion process, the addition of limonite does not negatively affect the hydrolysis process since the major composition of WSS; proteins and its enzyme, protease had slight or no significant difference between control WSS. Meanwhile in the acidogenesis stage, the variation of organic acids was reduced by the increased limonite concentrations, especially at 10% (w/v) of limonite. Besides, acetate accumulated in WSS with Aso limonite samples due to the inhibition of methane, except for 10% (w/v) of limonite. The accumulation of organic acids also could cause the pH level to be reduced, thus suppress the microbial activity for methanogenesis (Park et al. 2018). Therefore, acetic acid may be one of the factors reducing the pH of WSS during the fermentation with Aso limonite, which inhibited methane production. Additionally, the decrement of acetic acid have been reported to be indicators which reduce the emission of methane at the methanogenesis stage (Lee et al. 2011). This phenomenon of methane inhibition was shown in our study by the addition of 10% (w/v) of Aso limonite that recorded low acetic acid concentration and absent of other organic acids. Butyric and isobutyric acids, which have an unpleasant odour, decreased during the fermentation; it may indicate that limonite can remove not only malodorous hydrogen sulfide gas but also the odorous organic acids.

### Changes of microbial activity and community by Aso limonite

Based on qRT-PCR data, the number of archaeal population was lower in higher limonite concentrations. The result is reasonable to explain the low production of methane in the presence of Aso limonite because methane-producing microbes belong to the archaeal group. On the other hand, the number of bacteria reduced in WSS with the addition of Aso limonite, regardless of the concentration when compared to that in control WSS. Some of the bacterial community present in WSS may be unable to survive in the condition that pH was suddenly changed by the addition of Aso limonite.

Microbial community changes in WSS was evaluated by MiSeq analysis targeting 16S metagenomics. In control WSS, *Thiotrichales* found as an abundant population has been reported to be a sulphur-oxidizing bacterium in activated sludge (Zhang et al. 2017). *Thiobacterales* which is a sulphur-oxidizing bacterium and *Sphingobacteriales* which belongs to *Bacteroidetes* phylum are commonly found in the anaerobic digestion process of sludge (Bomberg et al. 2016). Whereas sulphur-oxidizing bacteria were found at a high ratio in the control WSS without limonite, interestingly, sulphate-reducing bacteria species were detected in the WSS in the presence of Aso limonite (*Desulfuromonadales* and *Desulfobacterales*). Theoretically, these two groups of bacteria will reduce the sulphate to produce hydrogen sulfide. Considering that no hydrogen sulfide was detected in this study (Fig. 1c), our verification that hydrogen sulfide adsorbed to limonite seems to be true. In addition, as reported by Siniscalchi et al. (2017), *Desulfuromonadales* and *Desulfobacterales* are also known as iron-reducing bacteria; therefore, the presence of these bacterial groups should be reasonable because Aso limonite is rich in iron. *Geobacter* species which is one of the dominant genus in *Desulfuromonadales* order is very well-known as an iron-reducing bacterium (Luef et al. 2013).

Moreover, in *Clostridiales* order, there are two dominant families present in limonite-added WSS which are *Clostridiaceae* and *Peptococcaceae*. *Clostridiaceae* is a common bacterial group found in sewage sludge during the anaerobic digestion process and is mainly responsible for the acidogenesis and acetogenesis stages whereas *Peptococcaceae* has been reported as a propionate-oxidizing bacterium (Imachi et al. 2002). This is supported by our result that a low amount of propionic acid was detected in the limonite-added WSS, specifically absent in WSS with higher Aso limonite addition (Fig. 3b). As another bacterial community, *Spirochaetales* was reported as one of the dominant iron-reducing bacteria and has been observed in abundance under iron-rich conditions (Baek et al. 2014). This is in agreement with our result that this population was higher in Aso limonite samples as compared to control WSS. Thus, bacterial community showing a characteristic change during the anaerobic digestion of WSS with or without Aso limonite could explain the differences in the production of intermediate products and methane production.

### Practical usage of Aso limonite for anaerobic digestion using WSS

In order to maintain the production of methane as the bioenergy source but reducing the hydrogen sulfide production, a separate system has been set up for WSS and limonite, which showed low hydrogen sulfide production. This result indicates that hydrogen sulfide was adsorbed by Aso limonite. The physical properties of limonite with large surface areas may provide more active sites for the adsorption of hydrogen sulfide. A study done by Zhou et al. (2016) using limonite ore as a desulfurizer in anaerobic digestion process showed an inhibitory effect of H_2_S production; however, there was no direct evidence that limonite adsorbed hydrogen sulfide in biogas. In this study, Aso limonite has the ability to mitigate the emissions of methane, hydrogen sulfide, and ammonia. The working mechanism of Aso limonite was related to its characteristics to adsorb hydrogen sulfide, to reduce ammonia by acidifying the process, and to inhibit the production of methane via the inactivation of methanogens. A contactless anaerobic digestion with Aso limonite may be useful as a means to remove hydrogen sulfide without inhibiting the production of methane.

## Data Availability

All raw sequences data are deposited into the NCBI Sequence Reads Archive (SRA) database under the Accession number of SRP072534.

## Abbreviations

WSS: Waste sewage sludge
VS: Volatile solid
DNA: Deoxyribonucleic acid
cDNA: Complementary deoxyribonucleic acid
RNA: Ribonucleic acid
rRNA: Ribosomal ribonucleic acid
qRT-PCR: Quantitative real-time polymerase chain reaction
